# Symmetrical peripheral gangrene: potential mechanisms and therapeutic approaches in severe COVID-19

**DOI:** 10.3389/fcvm.2023.1280625

**Published:** 2023-11-29

**Authors:** Manzhi Wang, Tao Sun, Liang Dong, Shengshi Huang, Ju Liu

**Affiliations:** ^1^ Post-doctoral Mobile Research Station, Shandong University of Traditional Chinese Medicine, Jinan, China; ^2^ Department of Hematology, The First Affiliated Hospital of Shandong First Medical University & Shandong Provincial Qianfoshan Hospital, Jinan, China; ^3^ Department of Orthopedic Surgery, The First Affiliated Hospital of Shandong First Medical University & Shandong Provincial Qianfoshan Hospital, Jinan, China; ^4^ Department of Respiratory and Critical Care Medicine, The First Affiliated Hospital of Shandong First Medical University & Shandong Provincial Qianfoshan Hospital, Jinan, China; ^5^ Institute of Microvascular Medicine, Medical Research Center, The First Affiliated Hospital of Shandong First Medical University & Shandong Provincial Qianfoshan Hospital, Jinan, China

**Keywords:** COVID-19, symmetrical peripheral gangrene, thrombotic microangiopathy, shock, disseminated intravascular coagulation

## Abstract

Symmetrical peripheral gangrene is a rare condition that is characterized by ischemic damage and tissue death (gangrene) in the extremities. Recent reports have shed light on SPG in patients with severe COVID-19. This condition presents with symmetrical cyanosis of the extremities and common COVID-19 symptoms and what the most frightening is within a few days, cutaneous necrosis occurred and patients died. Skin biopsy results have shown the presence of microthrombi in small vessels. The formation of SPG in COVID-19 patients results from immunothrombosis, endothelial dysfunction, and procoagulant platelets, leading to a hypercoagulation state and microvascular thrombosis. Thrombotic microangiopathy, shock, disseminated intravascular coagulation, and anticoagulant depletion promote the development of SPG in COVID-19. At the early stage, SPG patients with COVID-19 exhibit similar clinical manifestations. TMA causes early damage to microvasculature in SPG, and the shock state further exacerbates the ischemic injury due to local hypo-perfusion. The disturbed procoagulant-anticoagulant balance caused by DIC and anticoagulant depletion, combined with the pre-ischemic state brought on by TMA and shock, leads to the rapid formation of extensive microthrombi in the late stage of COVID-19 associated SPG. This review will delve into the clinical features, possible mechanisms, and potential therapeutic managements for COVID-19 associated SPG.

## 1. Introduction

The coronavirus responsible for the outbreak of COVID-19, known as severe acute respiratory syndrome coronavirus 2 (SARS-CoV-2), was initially detected in Wuhan city, Hubei province, China, in December 2019 (1). In early March 2020, the World Health Organization formally declared the global pandemic status of COVID-19. Despite primarily affecting the respiratory system, COVID-19 has been documented to cause a range of secondary effects, including myocarditis, acute kidney injury, hepatic dysfunction, and cutaneous manifestations.

The pandemic of COVID-19 has revealed an abnormal pattern of elevated thrombotic events, encompassing venous thromboembolism (VTE), arterial thrombosis, and thrombosis within the microvasculature. The underlying mechanisms behind the increased incidence of thrombosis in COVID-19 patients are not fully clarified but are thought to be related to a cytokine storm, hypoxia, endothelial dysfunction, hypercoagulability, and an increase in platelet activity (2–5).

Recent reports have described a unique cutaneous manifestation known as Symmetrical Peripheral Gangrene (SPG) in severe COVID-19 patients which present with symmetrical cyanosis of the extremities, along with symptoms such as fever, cough, hypoxemia, and hypotension. Unfortunately, all the COVID-19 patients eventually succumbed of SPG (6–8). Skin biopsy of patients with SPG reveals microthrombi in small vessels (6). This review aims to present comprehensive observations of the emerging clinical findings of SPG in COVID-19 patients to summarize the potential mechanisms, and describe the potential managements of COVID-19 associated SPG.

## 2. Case study of COVID-19 associated SPG

COVID-19 associated SPG presents with the formation of microthrombi and four main features: hypoxia, hypotension, DIC and AT depletion. Four cases of COVID-19 associated SPG have been reported (6–8) (Table 1). The patients consisted of three men and one woman, with ages ranging from 37 to 60. Of the two older patients, both had a history of hypertension. The presenting symptoms of the patients were fever, cough, and dyspnea. Upon admission, the patients were found to be hypoxic and hypotensive, with tachycardia and displayed cyanosis which was symmetrical at the upper and lower extremities. Polymerase chain reaction (PCR) tests, using the reverse transcription method for detecting SARS-CoV-2, yielded positive results from samples taken from nasopharyngeal and/or oropharyngeal swabs. Laboratory tests indicated notable extensions in both prothrombin time (PT) and activated partial thromboplastin time (APTT), alongside increased levels of D-dimer and fibrin degradation products (FDPs). Neutrophilic leukocytosis and lymphopenia were also observed. One patient with COVID-19-associated SPG had a low level of antithrombin III protein (ATIII) at 53% (normal range: 80%–120%) (7). During hospitalization, the cyanosis of the extremities rapidly progressed to cutaneous necrosis. Skin biopsy of the SPG patients revealed the presence of microthrombi in small vessels, with no signs of vasculitis or vasculopathy (6).

**Table 1:** Clinical manifestations, laboratory parameters, treatment, and outcome of four COVID-19 associated SPG patients.

	Patient 1	Patient 2	Patient 3	Patient 4
Age	37	42	78	60
Sex	Male	Male	Female	Male
Comorbidities	None	None	Hypertension	Hypertension
Clinical manifestations	Fever, cough, dyspnea, hypoxia (89%)	Fever, cough, dyspnea, hypoxia (84%)	Fever, cough, dyspnea, progressive confusion, hypoxia	NA
Pattern of gangrene	Symmetrical peripheral gangrene appearing in limb	Dry gangrene appearing in toes of both feet with ulceration	Cyanosis at the extremity, in particular at nose area, hands and feet fingers	Gangrene relating with toes of feet
BP (mmHg)	89/50	104/60	85/40	Shock
WBC (cells/ul)	14,000	12,860	15,560	NA
ALC (cells/ul)	1,050	990	490	NA
PLT (cells/ul)	56,000	87,640	58,000	NA
D-dimer(ng/ml)	11,562	9,356	>40,000	Elevated
DIC (ISTH)	Yes	Yes	Yes	NA
ATIII	NA	NA	53%	NA
Skin lesion pathology	Microthrombi	NA	NA	NA
Treatment received	Enoxaparin, methyl-prednisolone, supportive care	Enoxaparin, dexamethasone, supportive care	Antiviral agent, antibiotics, supportive care	Conservative supportive care
Outcomes	Death (after 12 days of hospitalization)	Death (after 10 days of hospitalization)	Death	Death (after 20 days of hospitalization)

BP, blood pressure; WBC, white blood cell; ALC, absolute lymphocyte count; PLT, platelets; DIC, disseminated intravascular coagulation; ATIII, antithrombin III protein; NA, not available.

## 3. The pathogenesis and etiologies of SPG

SPG is a rare complication that shows symmetrical distal ischemic damage and dry gangrene. The necrosis of SPG is predominantly acral and bilateral at least two locations including the toes, fingers, scrotum, and earlobes, without major vascular occlusive disease, while the term “purpura fulminans” (PF) refers to the occurrence the non-acral necrosis (9). Significant coldness, paleness, and bluish discoloration of the extremities are the precursors to suspect SPG, which advance rapidly to acrocyanosis and result in severe gangrene. Documented evidence shows a significant mortality rate, reaching up to 35%, and a substantial risk of multiple limb amputations, which occurs in up to 70% of those who survive SPG (10).

Sepsis is the underlying disorders of SPG, with some studies suggesting an estimated frequency of 2%–6% in sepsis (11). Coagulopathy induced by sepsis leading to disseminated intravascular coagulation (DIC), a severe fatal disorder, which is also recognized as one of the pathological features of SPG. DIC occurred in at least 90% of SPG patients (12), nonetheless, SPG develops in only a limited subset of patients who experience shock and DIC (13). Low-flow state, circulatory shock, were observed in SPG. In critically ill patients, the emergence of SPG typically lags, appearing no sooner than two days after the onset of shock and DIC, hinting at a temporal element in the development of this condition. In over 90% of cases, individuals with SPG experience “shock liver”, a precursor state to the development of ischemic necrosis in the limbs. The emergence of SPG is linked to a gradual decline in the concentrations of natural anticoagulants produced by the liver, especially antithrombin (AT) (14). Microthrombi in the capillary lumen, along with fibrin deposition and red blood cells extravasation, are observed in biopsy specimens of SPG patients (15). However, no evidence of vasculitis or inflammatory infiltrates was found on the vascular walls. Doppler ultrasonography of the peripheral arteries reveals that no large arteries are involved in the thrombosis (10).

The etiological factors leading to SPG include septic and non-septic factors, as shown in Table 2.

**Table 2:** Etiology of symmetrical peripheral gangrene.

Septic factors	Other factors
Bacterial	Cardiac disorders
Escherichia coli	Low output cardiac failure
Klebsiella pneumoniae	Third-degree atrioventricular block
Serratia marcescens	Ventricular tachycardia
Acinetobacter	Ventricular pseudoaneurysm
Pseudomonas	Myocardial infarction
Capnocytophaga canimorsus	Vasopressor drugs
Proteus mirabilis	Norpin
Salmonella paratyphi	Dopamin
Proteus vulgaris	Epinephrine
Pasteurella multocida	Noradrenaline
Dermabacter jinjuensis	Various autoimmune conditions
Neisseria meningitidis	Antiphospholipid syndrome
Streptococcus pneumoniae	Cryoglobulinemia
Staphylococcus aureus	Acquired hemolytic anemia
Mycobacterium tuberculosis	Systemic lupus erythematosus
Parasitic	Polymyalgia rheumatica
Plasmodium vivax	Malignancies
Plasmodium falciparum	Small cell lung cancer
Leptospira	Hodgkin's lymphoma
Viral	Drugs or poisons
Dengue virus	Phenylephrine
Rubeola virus	Sulphamezathine
Varicella zoster virus	Penicillin
Human immunodeficiency virus	Propylthiouracil
	Warfarin
	Snake venom
	Therapy-related
	Extracorporeal membrane oxygenation
	Idiopathic SPG

## 4. Exploring the manifestations and pathophysiology of COVID-19 and SPG

### 4.1. The early damage to microvasculature of SPG is potentially caused by COVID-19-induced thrombotic microangiopathy

Thrombotic microangiopathy (TMA), a frequently observed form of microvascular thrombosis, constitutes a pathological characteristic of COVID-19 (5, 16). The presence of retiform purpura and livedo racemose, which are relatively infrequent dermatological manifestations of COVID-19, are distinguished by pauci-inflammatory TMA findings in skin biopsy outcomes (17, 18). All hospitalized patients diagnosed with retiform purpura exhibited acute respiratory distress syndrome in 82% of cases, with bilateral lesions on their extremities and buttocks showing varying degrees of severity (17, 19). Some SPG patients exhibit additional symptoms such as neurological complications, renal dysfunction, thrombocytopenia, and significant increase in lactate dehydrogenase (LDH) (6, 7). Retiform purpura and livedo racemose-like lesions have often been seen in patients with apparent coagulopathy, therefore, based on the comparable clinical presentations and pathological alterations, SPG may represent a more severe and advanced type of retiform purpura/livedo racemose in COVID-19.

### 4.2. Septic shock is the potential cause of SPG in COVID-19 patients

The SARS-CoV-2 virus, being an infectious pathogen, has the potential to induce sepsis or septic shock in individuals with COVID-19. However, the prevalence of shock in adult COVID-19 patients varies greatly (ranging from 1% to 35%), and is contingent upon the population being researched, the disease severity, and the definition of shock (20, 21). Sepsis or septic shock contributes to persistent vasodilation, consequently causing hypotension and hypoxia (22).

All SPG patients in COVID-19 all had low blood pressures and three of them developed shock. The shock state exacerbates the already present ischemic injury by causing local hypoperfusion, leading to multiple organ failures, including liver dysfunction (7).

### 4.3. Disturbed procoagulant–anticoagulant balance in COVID-19 patients induces rapid formation of extensive microthrombi

The significance of DIC in SPG is supported by pathological studies revealing noninflammatory fibrin microthrombi deposits in small vessels (23). At the decompensated stage, excessively activated coagulation process leads to microthrombus generation and the depletion of clotting factors. According to the DIC score of the International Society on Thrombosis and Haemostasi, APTT and PT were prolonged and the levels of FDPs and D-dimer are dramatically elevated, while the platelet numbers and fibrinogen levels are already decreased (24).

DIC and anticoagulant depletion greatly disturb the procoagulant–anticoagulant balance and lead to the rapid formation of extensive microthrombi at the late stage of COVID-19 associated SPG. DIC has been recognized in the advanced stage of severe COVID-19. During hospitalization, the criteria of DIC were met by 71.4% of non-survivors, while only 0.6% of survivors (24).

In severe COVID-19 patients, endothelial dysfunction, decreased liver synthesis, degradation through proteolytic cleavage enzymes of neutrophils, and excessive consumption due to DIC contribute to the anticoagulant depletion. Goshua and colleagues demonstrated that an elevation in the shedding of thrombomodulin from endothelial cells in patients with acute COVID-19 has been observed (25), which impairs the anticoagulant activity of protein C (PC). Another crucial component of the natural anti-coagulation system, ATIII, is frequently deficient in severe COVID-19, with at least 25% of patients showing ATIII deficiency (26). ATIII level was significantly lower in non-survivors than survivors in COVID-19 (27). Patient 3 presented with low ATIII level (7). In addition, elevated levels of plasminogen activating inhibitor-1 (PAI-1) combined with hypofibrinolysis were found in COVID-19 patients (28). The hypofibrinolytic state suggests more extensive microthrombosis and less frequent hemorrhagic events in COVID-19 related DIC.

## 5. SARS-COV-2 infection and SPG: insight into underlying molecular machanism

### 5.1. Infection with SARS-CoV-2 causes direct damage to vascular endothelial cells, potentially causing a procoagulant state

SARS-CoV-2 targets and invades cells lining the respiratory tract, type II pneumocytes in the alveoli, and cells comprising the vascular endothelium by binding to angiotensin-converting enzyme 2 (ACE2) (29). COVID-19 cases reported by Varga et al. found the evidence that SARS-CoV-2 directly infects vascular endothelial cells (30). The integrity of the vascular endothelium is closely related to the hypercoagulable state. Intact endothelium secretes amounts of anticoagulant factors to prevent platelet activation and coagulation, such as nitric oxide, inhibitors of the tissue factor pathway such as TFPI, the coagulation-regulatory protein thrombomodulin, and the endothelial protein C receptor (EPCR). Direct infection of the endothelium by SARS-CoV-2 potentially damages endothelial surface, suppressing the expression of TFPI, TM, and EPCR, which is thought to the leading cause of procoagulant state in COVID-19 patients (31). In an autopsy study of 26 patients who died from COVID-19, researchers explored how SARS-CoV-2 infection damaged human kidneys. Results showed glomerular fibrin thrombosis in COVID-19 patients (32), which means that the SARS-CoV-2 infection may lead to a breakdown of the ECs barrier.

### 5.2. Activation of immune system caused by SARS-CoV-2 infection induces microvascular thrombosis associated SPG

**Figure 1:**
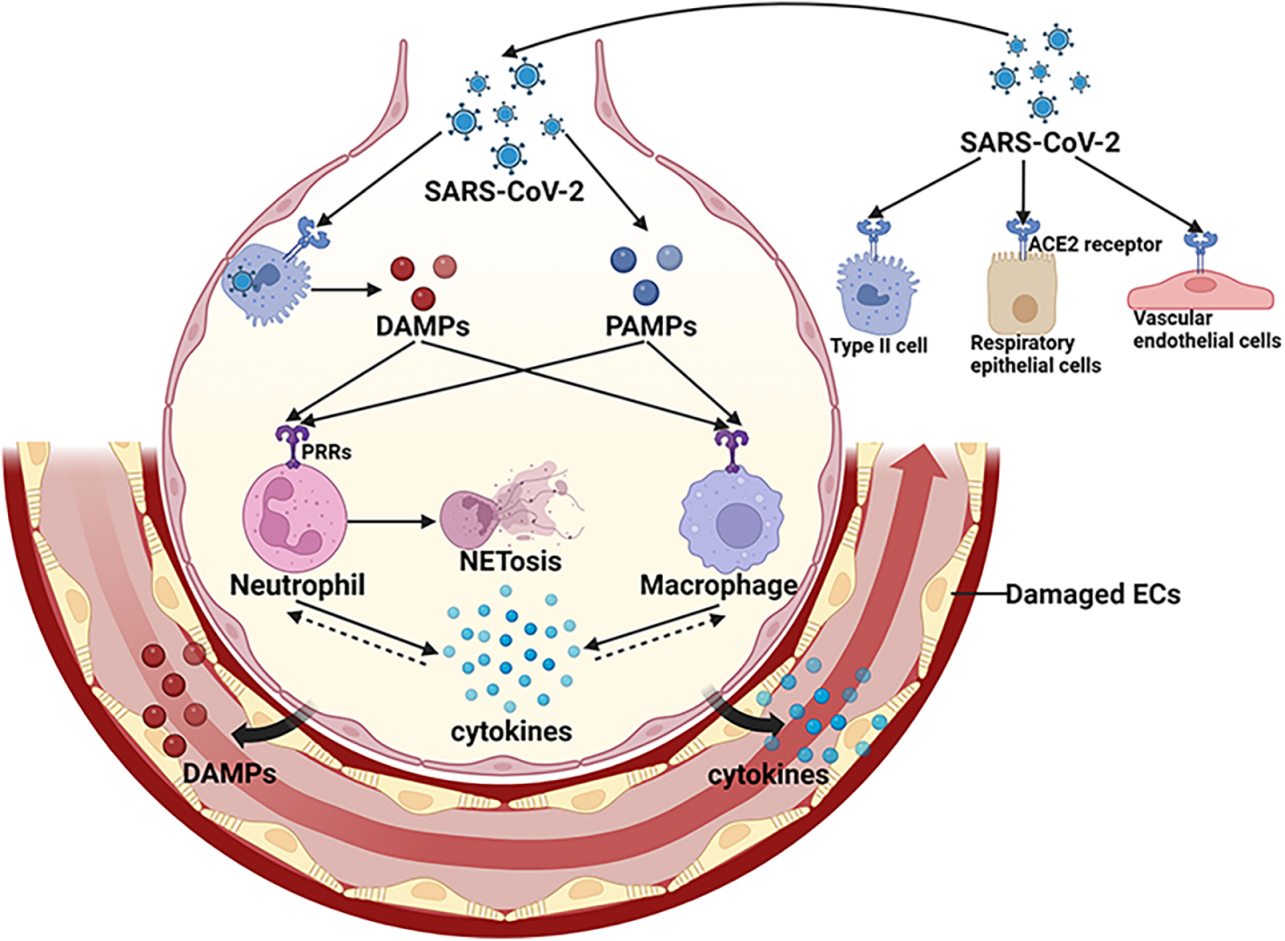
Activation of local innate immune system in COVID-19. SARS-CoV-2 infects respiratory epithelial cells, alveolar type II cells and vascular endothelial cells by binding to ACE2. The PAMPs of SARS-CoV-2 and DAMPs released by infected host cells activate immune cells through PRRs signaling pathways, resulting in transcription of various cytokines. These cytokines cause the recruitment of immune cells, aggravating the local inflammatory response. Virus proteins, DAMPs and cytokines can induce excessive release of NETs. In severe cases, viral infection and over-activated immune systems cause further damage to pulmonary vasculature, leading to a breakdown of ECs barrier and the leakage of pro-inflammatory mediators, such as DAMPS and cytokines.SARS-CoV-2, severe acute respiratory distress syndrome-associated coronavirus-2; DAMPs, damage associated molecular patterns; PAMPs, pathogen associated molecular patterns; PRRs, pattern recognition receptors; ACE2, angiotensin converting enzyme-2; ECs, endothelial cells; NET, neutrophil extracellular trap. (Created with BioRender.com).

Microvascular thrombotic events in COVID-19, usually characterized as immunothrombosis, are induced by the activated systemic immune systems, such as cytokine storm, neutrophil extracellular traps (NETs), and activated complementary system (5, 33). SARS-CoV-2 replication and viral particles released by infected host cells cause tissue damages in the lung (5). The pathogen associated molecular patterns (PAMPs) of SARS-CoV-2, including its spike glycoprotein and single-stranded RNA, along with host-derived molecular markers like high-mobility group box 1 (HMGB1) from infected cells, trigger immune responses via pattern recognition receptor (PRR) signaling cascades. This activation results in the synthesis and dissemination of chemokines and pro-inflammatory cytokines, encompassing tumor necrosis factor (TNF), interleukin-1β (IL-1β), IL-2 receptor (IL-2R), IL-6, and IL-8 (34). These chemokines cause the recruitment of neutrophils, macrophages and CD4 positive T lymphocytes in lung tissue (35), aggravating the local inflammatory response. Virus proteins, HMGB1 and cytokines can induce excessive release of NETs (36), which activate the oxygen species (ROS) formation (37). In severe COVID-19 patients, SARS-CoV-2 infection, NET proteins and ROS cause the pulmonary capillary destruction and leakage of proinflammatory mediators, such as HMGB1 and inflammatory cytokines (38–40) (Figure 1). DAMPs and cytokines in circulation further activate the systemic immune systems (41) and trigger intravascular thrombus formation through inducing the tissue factor (TF) expression on vascular ECs and monocytes (42).

The cytokine storm gives rise to lymphopenia which is prevalent in patients with COVID-19 and exists in two cases of COVID-19 associated SPG (6, 7). Lymphopenia impairs the function of the immune system to produce antibodies against virus-specific antigens, as well as reducing the generation of interferon gamma (IFN- γ) by the CD4+ T cells (43). Impaired clearance of the SARS-CoV-2 leads to the viremia in the circulation (44). High plasma levels of SARS-CoV-2 induce the elevation of cytokines such as IL-6 and enhance severe COVID-19 progression (45). The discovery of elevated levels of cytokines, like IL-6, in severe COVID-19 patients could be both a result and a cause of viremia, creating a potentially hazardous cycle that perpetuates itself.

The amount of Circulating NET-forming neutrophil subset (immunotype DEspR+CD11b+) and NETosis induction levels are positively related with the progression, severity, and duration of acute COVID-19 (46–48). The levels of NETosis initiation showed a significant correlation with platelet activation indicators and blood clotting-related factors (47, 49). NETs components cause the degradation of TFPI via neutrophil elastase during thrombotic complications (49), thus activating the coagulation cascade TF pathway (50). The initiation of the intrinsic blood clotting mechanism occurs when NETs carrying a negative charge form a direct association with, and set into action, the coagulation factor FXII. NETs additionally adhere to the Von Willebrand Factor (vWF), offering a foundation for platelets to attach (49). Complexes formed by NETs and platelets serve as frameworks that facilitate the attachment of other molecules promoting coagulation, like fibronectin and fibrinogen. Consequently, these complexes capture free-floating blood cells and encourage their clustering, leading to the creation of blood clots and the blockage of blood vessels (51). Elements of NETs, such as histones and dsDNA, contribute to the advancement of thrombosis by enhancing the density, stiffness, and resilience of fibrin strands while obstructing the breakdown of fibrin (52).

By directly interacting with Mannan-binding lectin-associated serine protease-2 (MASP-2), SARS-CoV-2 activates the lectin complement pathway and causes ECs damage through membrane attack complex (MAC) (53). MASPs contribute to thrombus formation by cleaving prothrombin to form activated thrombin (54). Furthermore, the activated complement pathway releases C5a which increases TF activity via C5a receptors on neutrophils and endothelial cells (55).

### 5.3. Endothelial injury and platelets activation induced by COVID-19 contributes to microvascular thrombosis associated SPG

Activated systemic immune systems, viremia and hypoxemia in severe COVID-19 exert cytotoxic effects on ECs (5, 30). The injured ECs upregulate the expression of adhesion proteins, including intercellular adhesion molecule-1 (ICAM1) and vascular cell adhesion molecule-1 (VCAM1), chemoattractants like monocyte chemoattractant protein-1 (MCP-1), and inflammatory mediators such as interleukins IL-1 and IL-6 (56), which aggravates systemic inflammatory reactions and immunothrombosis (57). vWF release induced by endothelial injury mediates platelet aggregation through the binding with glycoprotein Ib-IX-V complex and integrin αIIbβ_3_ on platelets (58). Under typical conditions, enzymes present in the plasma like A disintegrin and metalloproteinase with thrombospondin motifs 13 (ADAMTS-13) are responsible for breaking down vWF multimers (59). However, in COVID-19, peptidyl arginine deiminase-4 (PAD4), an enzyme expressed in NETs, changes the structure of ADAMTS-13 and reduces the degrading ability of ADAMTS-13 (60). The increase in the vWF/ADAMTS-13 ratio, which reflects the thrombogenicity of the blood, suggests that hypercoagulable state in COVID-19 patients (61). In COVID-19 cases, the concentration of soluble P-selectin was markedly higher in patients those in the ICU as opposed to those not or healthy individuals (25, 62). P-selectin overexpressed by endothelial injury interacts with its ligand P-selectin glycoprotein ligand-1 (PSGL-1) on almost all leukocytes (63) and the glycoprotein Ibα (GPIbα) receptor on platelets, which facilitates the attachment of leukocytes and platelets to the injured regions of the endothelium through activation of the PSGL-1 signaling in leukocytes and the GPIb signaling in platelets (64, 65). Furthermore, endothelial injury upregulates the expression of TF (66) which combines with coagulation factor VII (FVII) and promotes extrinsic pathway.

**Figure 2:**
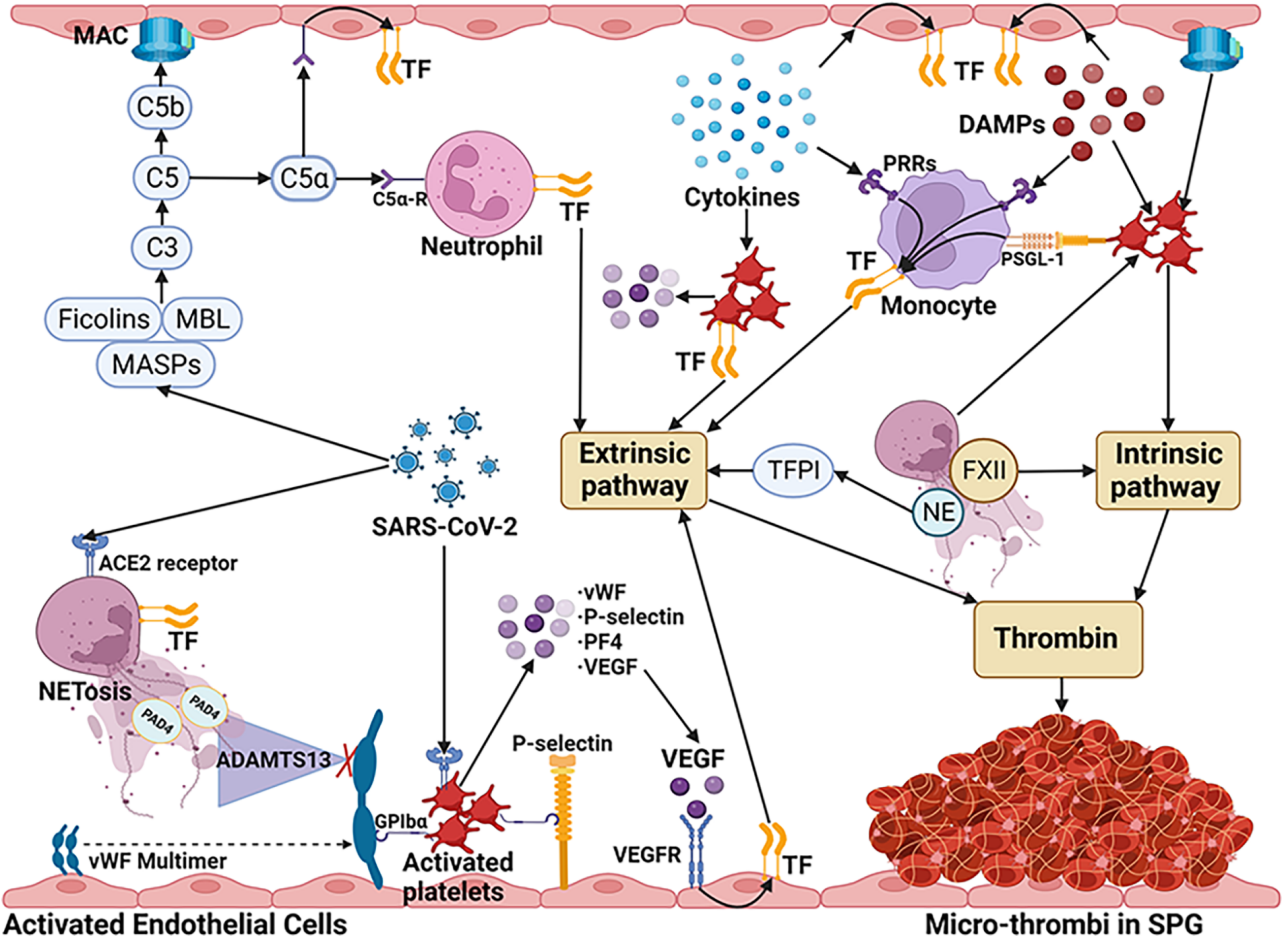
The main mechanisms of microvascular thrombosis in COVID-19 associated SPG. SARS-CoV-2 stimulates neutrophils to release NETs via ACE2 and activates the NET/TFPI/TF and NET/FXII pathways. By directly binding to MASP-2, SARS-CoV-2 can activate the lectin complement pathway and cause ECs damage through MAC. C5a which is released by the activated complement pathway increases TF activity via C5a receptors on neutrophils and ECs. SARS-CoV-2 also can bind to ACE2 on platelets, leading to the release of granules, such as vWF, P-selectin, PF4, and VEGF. VEGF triggers ECs to upregulate the expression of TF. Due to the interaction with PAD4 released by NETs, the degrading ability of ADAMTS-13 decreased, aggravating the increase of vWF. vWF multimers and P-selectin released by activated ECs, lead to aggregation of platelets by GPIbα receptor. P-selectin on activated platelets induce TF expression of monocytes by PSGL-1 signaling pathway. DAMPs and cytokines inducing the expression of TF on monocytes and vascular ECs. The cytokines also can induce the TF expression and the granules release of platelets. In addition, NETs, DAMPs (such as HMGB1), and complementary system also contribute to the activation of platelets. The coagulation cascades are initiated mainly by TF through the extrinsic pathway. Furthermore, activated platelets can directly activate the intrinsic pathway by assembling coagulation factors on their surface. These mechanisms ultimately lead to the unchecked generation of thrombin, resulting in micro-thrombus formation in COVID-19 associated SPG. SARS-CoV-2, severe acute respiratory distress syndrome-associated coronavirus-2; NETs, neutrophil extracellular traps; ACE2, angiotensin converting enzyme-2; TFPI, tissue factor pathway inhibitor; FXII, factor XII; MASP, Mannan-binding lectin-associated serine protease; ECs, endothelial cells; MAC, membrane attack complex; TF, tissue factor; PF4, platelet factor 4; VEGF, vascular endothelial growth factor; DAMPs, damage associated molecular patterns; HMGB1, high mobility group box 1; PAD4, peptidylarginine deiminase; ADAMTS-13, A disintegrin and metalloproteinase with thrombospondin motifs 13; vWF, von Willebrand factor; GPIbα, glycoprotein Ibα; PSGL-1, P-selectin glycoprotein ligand 1; PRRs, pattern recognition receptors; MBL, mannan-binding lectin. (Created with BioRender.com).

Thrombocytopenia was revealed in the patients with COVID-19 associated SPG (6–8). In COVID-19 patients, a significant association between low platelet counts and increased disease severity as well as high mortality rates has been reported (67, 68). In individuals who succumbed to COVID-19, autopsies revealed a substantial presence of megakaryocytes and clots abundant in platelets within the heart, pulmonary, and renal tissues (69, 70). Platelets release procoagulant factors in response to the cytokine storm (71). Furthermore, the engagement between NETs and platelets, coupled with platelet activation driven by the HMGB1-Toll-like receptor (TLR)-4 pathway, impact the initiation of the coagulation process, resulting in the widespread generation of microthrombi (36). Platelets may also be activated by the incorporation of the C5b-9 complex into their surface (72), the binding of C1q to its corresponding receptor on the platelet membrane (73), and their responsiveness to component C3 (74). It has been reported that human and mouse platelets express ACE2, to which the spike protein of SARS-CoV-2 binds, leading to the release of vWF from α- and dense granules (75). Activated platelets express TF and P-selectin on their membranes, which promotes their binding to leukocytes and endothelial cells, enhancing the expression of TF by activating the nuclear factor kappa-light-chain-enhancer of activated B cells (NF-κB) pathway (76). As a coagulation factor, PF4 released by activated platelets neutralizes heparin-like molecules on the endothelial surface of blood vessels. Increased PF4 levels and enhanced platelet-neutrophil aggregates were found in COVID-19 patients (77). Upon activation, platelets discharge vascular endothelial growth factor (VEGF), which triggers endothelial cells to upregulate the expression of TF (66). Additionally, activated platelets exhibit catalytic activities by assembling coagulation factors on their surface (78).The main mechanisms of microvascular thrombosis in COVID-19 associated SPG were summarized in Figure 2.

## 6. Potential approaches for managing thrombotic events in patients with COVID-19 associated SPG

**Figure 3:**
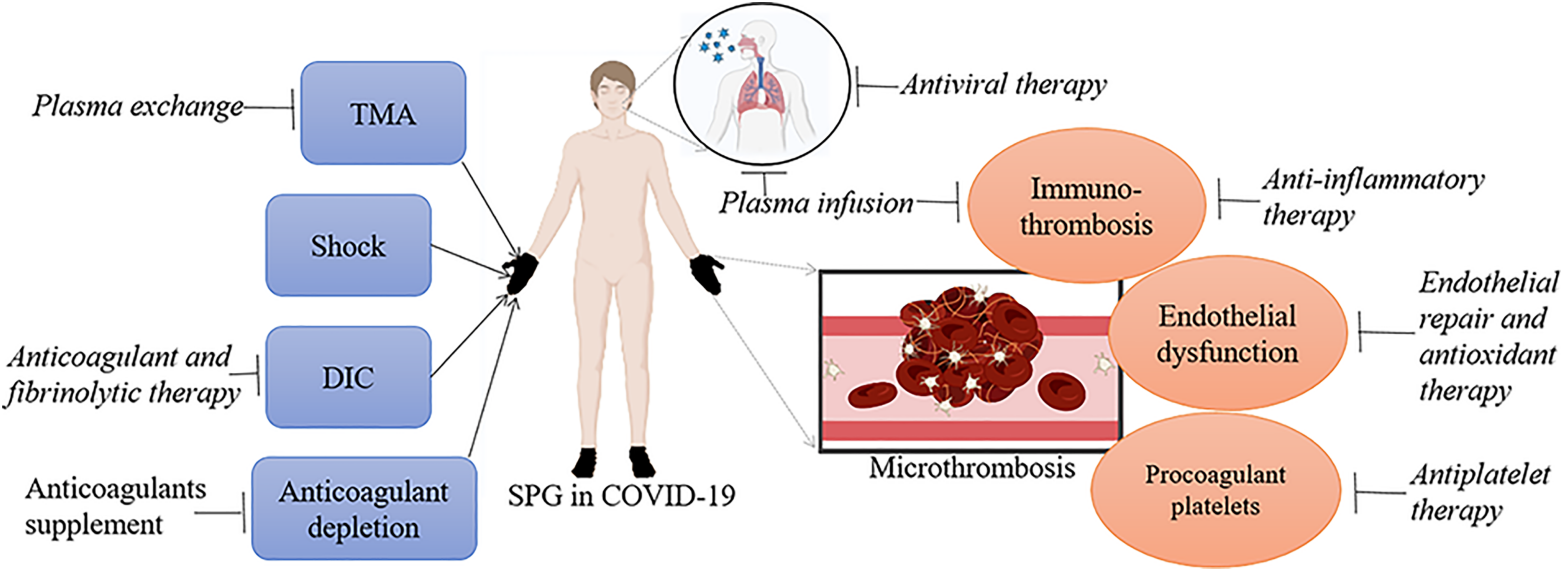
The target point for each therapy in COVID-19 associated SPG. TMA, shock, DIC and anticoagulant depletion contribute to the formation of SPG in COVID-19. Plasma exchange pointing at TMA, anticoagulant and fibrinolytic therapy pointing at DIC, and anticoagulants supplement pointing at anticoagulant depletion may do favor to these patients. Immunothrombosis, endothelial dysfunction and procoagulant platelets are possible molecular mechanism of COVID-19 associated microthrombosis. Anti-inflammatory therapy and plasma infusion pointing at immune disorders, endothelial repair and antioxidant therapy pointing at endothelial dysfunction, and antiplatelet therapy pointing at procoagulant platelets may be helpful for SPG patients with COVID-19. Finally, SARS-CoV-2 is the main trigger for COVID-19 associated SPG, antiviral therapy can lower the virus loads and reduce the occurrence of COVID-19 related complications, especially microthrombosis.

In the realm of managing COVID-19 associated SPG, a multifaceted therapeutic approach is crucial due to the complex pathophysiology of the disease. The treatment strategies encompass a broad spectrum, ranging from targeting the virus itself to addressing the systemic effects induced by the infection. This comprehensive review elucidates the multifarious treatment options (Figure 3), underlining the necessity of a tailored therapeutic regimen based on individual patient profiles and disease severity in COVID-19 associated SPG.

### 6.1. Antiviral therapy

During the early phase of infection, when the viral load of SARS-CoV-2 is elevated and the patient exhibits mild to moderate symptoms, medications aimed at inhibiting viral replication may prove more beneficial, as the adaptive immune system has yet to mount a sufficient response. Administering antiviral medications promptly increases their potential effectiveness, particularly for individuals at elevated risk (79–82). Now the common used antiviral drugs include protease inhibitors (like Paxlovid) and RNA-dependent RNA polymerase (RdRp) blockers (like Remdesivir and Molnupiravir). Paxlovid, a combined formulation of Nirmatrelvir and Ritonavir tablets, inhibits the SARS-CoV-2's 3-CL protease during the proteolysis stage, thereby preventing the virus from replicating (83). It was formulated for the management and post-exposure prevention of COVID-19 in patients who are more likely to develop severe symptoms (84). Molnupiravir, a ribonucleoside prodrug of N-hydroxycytidine effectively blocks the RdRp of SARS-CoV-2, thereby preventing the virus's ability to transcribe and replicate its genetic material (85). When administered within 5 days of symptom onset, it has the potential to lower the likelihood of hospitalization and fatality in individuals with COVID-19 who are more susceptible to progressing to a severe form of the disease (86). Remdesivir is another RdRp blocker (87). Being the initial drug to receive FDA approval for COVID-19 treatment, Remdesivir demonstrates strong binding capabilities with the SARS-CoV-2 spike, ACE2, and transmembrane protease serine2 (TMPRSS2), suggesting its potential to block the entry of the virus (88). The research trial indicated that Remdesivir successfully reduced the duration of recovery and hindered the advancement of respiratory illness in patients with COVID-19 (89). By effectively reducing viral load and inhibiting virus entry into host cells, the following inflammatory response, injury to the endothelium, and initiation of coagulation will subsequently diminish, leading to the reduced COVID-19 associated complications, especially the occurrence of thrombosis (Figure 3).

### 6.2. Anticoagulant therapy

Heparin or low-molecular-weight heparin (LMWH) is recommended as prioritized anticoagulant due to its monitorable anticoagulant effect. Additionally, liver dysfunction and renal failure does not affect heparin clearance. Besides its anticoagulant properties, heparin has anti-inflammatory effects (90). A recent meta-analysis found that patients with sepsis, septic shock, and infection associated DIC those who received heparin (compared with usual care or placebo) had a 12% decrease in mortality rate (90). Fondaparinux sodium, a selectively synthesized inhibitor of factor Xa, is authorized for use in both preventing and treating VTE incidents in patients who are acutely unwell (including those with COVID-19 or cancer) as well as individuals undergoing surgical procedures (91). Additionally, it is applicable for use in cases of acute coronary syndromes, bariatric surgical procedures, heparin-induced thrombocytopenia (HIT) (92), and vaccine-induced immune thrombotic thrombocytopenia (VITT) (93). The incidence of venous thromboembolism (VTE) at 6.5% compared to 13.5% (*P* = 0.36), and bleeding events at 6.5% vs. 4.1% (*P* = 0.68), did not significantly differ between the group of COVID-19 patients administered fondaparinux and the group given enoxaparin treatment (94). Moreover, fondaparinux presents several clinical benefits for individuals diagnosed with COVID-19, based on its possible antiviral (95) and anti-inflammatory effects (96). In this review, despite receiving appropriate treatments, such as enoxaparin, glucocorticoids, and antiviral agents, all COVID-19 patients suffered from SPG succumbed of ARDS or multiple organ failure within a few days of hospitalization (6–8). In addition, anticoagulant therapy is controversial in terms of dose and intensity. Accordingly, we need to identify the early signals of COVID-19 associated SPG, such as symmetrical purpura and TMA related indexes, and optimize the timing and dose of anticoagulant therapy (97).

In a clinical trial of patients with severe sepsis, despite the fact that the death rate was lower in AT-treated individuals with DIC who were not administered heparin (98), high-dose AT concentrates did not improve the total mortality (99). In addition, after a large-scale, randomized trial failing to demonstrate an improvement in survivals of patients with septic shock, recombinant activated protein C was removed from the candidate drugs (100). Employing rTFPI as part of therapeutic approaches targeting the fibrinolysis system might be effective against endothelial damage, inflammation, NETosis, and coagulation disorders induced by SARS-CoV-2 (52).

### 6.3. Anti-inflammatory therapy

Corticosteroids, recognized medications for addressing inflammation and autoimmune disorders, connect with nuclear receptors, subsequently diminishing the secretion of proinflammatory cytokines (101). Across various laboratory and live organism studies, steroids prompt a decrease in the formation of NETs (102). Steroids additionally diminish the secretion of HMGB1 and its engagement with TLR4 (103). The evidence regarding their application in COVID-19 remains inconclusive (104–107). Recent findings suggested that administering methylprednisolone in the early stages for a short duration is linked to improved clinical results in patients experiencing severe COVID-19 pneumonia, and such treatment should be contemplated prior to the onset of ARDS (108, 109).

Considering the presence of cytokine storm and inflammatory response in sepsis and coagulopathy of COVID-19 patients (38, 39, 110), IL-6 is identified as a potential drug target. RECOVERY trial results indicate that tocilizumab (anti-IL-6 drug), along with corticosteroids, improved the survival in patients with COVID-19 who had hypoxia and systemic symptoms (111). Janus kinase (JAK) inhibitor has shown therapeutic potential in severe COVID-19 by suppressing inflammatory pathway (112) and should be considered in the treatment of SPG patients.

NETs play a vital role in immunothrombosis after SARS-CoV-2 infection. Continued efforts that prevent NET release could potentially be a successful tactic in managing COVID-19.associated coagulopathy. The release of NETs can be controlled by curbing the generation of ROS and hypochlorous acid (HOCl). This can be achieved either by suppressing NADPH oxidase and/or myeloperoxidase (MPO) activity or by adding antioxidants (113). For instance, both metformin and diphenyleneiodonium (DPI) have the capacity to reduce NETosis, owing to their potential to inhibit the activity of NADPH oxidase and reduce the production of reactive oxygen species from mitochondria (114, 115). Likewise, augmenting with various compounds that neutralize ROS, such as resveratrol, flavonoids, and N-acetylcysteine, can prove efficacious in minimizing the discharge of NETs (116–119). Moreover, individuals with severe or critical COVID-19 exhibited notably reduced levels of NETosis following treatment with dexamethasone (47).

Therapeutic drugs which inhibit the complement process potentially inhibit the process of sepsis and the overactive inflammatory reaction in COVID-19 (18, 110). For example, in the case of COVID-19 patients with severe pneumonia, Diurno et al. noted that after initiating treatment with eculizumab, a complement system inhibitor, there was observable progress in clinical symptoms, lung lesions identified via CT scans, and laboratory test results within the first 48 h (120).

### 6.4. Endothelial repair and antioxidant therapy

COVID-19 outcomes are adversely affected by preexisting endothelial dysfunction, which is associated with sex, smoking, metabolic syndrome, and existing cardiovascular diseases (121). Severe COVID-19 results in endothelial dysfunction, leading to a shift towards a pro-coagulant state with increased vasoconstriction and inflammation (56). In addition, endothelial injury is a core pathogenesis and the therapeutic target of TMA (122, 123). Agents with endothelial cell-modifying effects, for example, phosphodiesterase inhibitors (PDEi) (124) potentially have therapeutic effect in SPG patients with severe COVID-19.

N-acetylcysteine (NAC), as an antioxidant, increases the biosynthesis of glutathione and reduces the generation of reactive oxygen species (ROS) (125). In recent years, NAC which protects endothelial cells free from ROS attack has been utilized in cases of thrombotic thrombocytopenic purpura and in transplant-associated thrombotic microangiopathy (126, 127). Research showed that NAC protects COVID-19 patients from oxidative stress-mediated endothelial damage (128).

### 6.5. Antiplatelet and fibrinolytic therapy

Clinically, dipyridamole (DIP), a drug that possesses anti-platelet aggregation properties, has been linked to elevated platelet counts and decreased levels of D-dimer. In both animal and *in vitro* research, DIP has been shown to inhibit the replication of SARS-CoV-2 and stimulate a type I interferon (IFN) response (129). Previous research has reported that DIP has protective effect against the aggravation of endotoxin induced DIC in experimental animals (130). The impact of aspirin use, another anti-platelet aggregation drug, has been examined in cases of COVID-19 infection accompanied by ARDS. Nonetheless, the findings have been varied, with certain studies indicating benefits while others have not shown positive outcomes (131–134). To ascertain the definitive efficacy of antiplatelet therapy in COVID-19, randomized controlled trials involving varied patient groups are necessary.

Wang J et al. documented instances of three individuals with COVID-19-induced ARDS who underwent treatment with Alteplase, an intravenous tissue-type plasminogen activator (tPA). Their findings revealed an initial improvement of PaO_2_/FiO_2_ ratio ranging from 38% to 100% in all cases. In addition, the administration of tPA systemically was linked to a decrease in mortality [47.6% (tPA) vs. 71.0% (no tPA)] for COVID-19 patients who had a refractory PaO_2_/FiO_2_ of less than 60 mmHg (135). Nonetheless, the widespread administration of fibrinolytic agents carries the hazard of possibly lethal bleeding events. Indeed, nearly 7% patients subjected to such agents require transfusion of blood products, while approximately 1% succumb to the effects of hemorrhages (136). Administering the treatment locally through nebulization presents a compelling alternative, possibly offering enhanced effectiveness while minimizing the risk of bleeding. The nebulized form of recombinant tPA may aid in promoting localized clot breakdown within the alveolar space and enhance oxygen levels (137). The improvement of hypoxia status is also beneficial for improving COVID-19 related lung injury and other complications. Finally, enhanced-fibrinolytic-type DIC occurs at advanced stage of COVID-19, which means fibrinolytic therapy is only effective in the early stages of COVID-19 (138). Therefore, early recognition of SPG before the onset of enhanced-fibrinolytic-type DIC is critical.

### 6.6. Plasma exchange

In patients with COVID-19 suffering from SPG, plasma exchange (PEX) may serve as a therapeutic intervention, ideally initiated within the first 48 h after shock liver onset (14). PEX operates by a mechanism akin to that seen in other TMAs, wherein it clears detrimental substances such as endotoxins and inflammatory cytokines while replenishing depleted components of the coagulation system, including natural anticoagulants and cofactors, thus inhibiting fibrin production throughout various stages of the coagulation pathway (139).

### 6.7. Recovered plasma and intravenous immunoglobulins

Administering serum from individuals who have healed and tested PCR-negative, enriched with IgG antibodies against SARS-CoV-2 [also termed hyperimmune IgG-containing plasma (HIgCP)], offers a treatment strategy for recently infected individuals, drawing from past learnings associated with various viral ailments. Utilizing HIgCP could offer therapeutic advantages for the management or prevention of ARDS caused by SARS-CoV-2 (140, 141). Nonetheless, the requirement for a compatible blood type between the donor and recipient, coupled with the potential danger of contracting additional viral infections, renders HIgCP less apt for widespread use. Intravenous immunoglobulins (IVIg) have been influential in adjusting immune reactions. IVIg reduces NET formation (142), cytokine and DAMP production (143). Moreover, IVIg offers protection from cell death initiated by HMGB1, influencing the expression of TLR and RAGE (144). The efficacy of IVIG treatment in managing COVID-19 is still a matter of debate. The outcomes of a meta-analysis suggested that IVIg treatment did not have a discernible impact on either the death rate or the duration of hospitalization (145). While other study showed that IVIG might decrease the death rate in comparison to the control group in severely ill COVID-19 individuals (146). So IVIG has shown to be clinically effective on critical ill patients which indicated its use in SPG patients with severe COVID-19.

### 6.8. Vascular dilation therapy

Cases of peripheral gangrene have been documented subsequent to the use of several vasoconstrictive medications such as vasopressin, dopamine, and noradrenaline, and traditionally, it occurs after consuming ergot (147–151). Vasodilators such as prostaglandin (152), epoprostenol (150, 153) and iloprost (154) may minimize tissue loss with regression of necrotic lesions in SPG, especially applied in early stage (150). However, the use of vasodilators is mainly reported in case reports, with the combination of other treatments, such as tissue plasminogen activator (153) and heparin (154). To verify their healing impact on SPG, rigorous clinical studies must be conducted.

## 7. Conclusion

SPG is a frequently overlooked complication of severe COVID-19 that arises from a systemic disorder rather than a localized vascular disease. COVID-19 associated SPG presented with formation of microthrombi and four main features: hypoxia, hypotension, DIC and AT depletion. Immunothrombosis, endothelial dysfunction and procoagulant platelets contribute to the formation of microvascular thrombosis. TMA, circulatory shock, DIC and anticoagulant depletion enhance the development of COVID-19 associated SPG. Ultimately, SPG occurs in COVID-19 due to the procoagulant-anticoagulant imbalance caused by the greatly disturbed microvasculature at risk, the DIC state, and anticoagulant depletion. Managing strategies are to treat patients suffering SPG from the perspective of anticoagulation benefiting or without affecting the treatment of COVID-19. Searching for early indicators from the progression of COVID-19 are still the keys to manage SPG.
